# Controversies in the Application of AI in Radiology—Is There Medico-Legal Support? Aspects from Romanian Practice

**DOI:** 10.3390/diagnostics15020230

**Published:** 2025-01-20

**Authors:** Ana-Maria Ungureanu, Sergiu-Ciprian Matei, Daniel Malita

**Affiliations:** 1 Department XV, Clinic of Radiology and Medical Imaging, “VictorBabes” University of Medicine and Pharmacy, Eftimie Murgu Square, No. 2, 300041 Timisoara, Romania; ungureanu.ana-maria@umft.ro (A.-M.U.); malita.daniel@umft.ro (D.M.); 2 Department of Radiology and Medical Imaging, “Pius Brinzeu” Emergency County Hospital, 300723 Timisoara, Romania; 3 Abdominal Surgery and Phlebology Research Center, Victor Babeș University of Medicine and Pharmacy, 300041 Timișoara, Romania

**Keywords:** artificial intelligence, liability, malpractice, misdiagnosis, medico-legal, radiological diagnosis

## Abstract

**Background/Objectives**: Artificial intelligence (AI) is gaining an increasing amount of influence in various fields, including medicine. In radiology, where diagnoses are based on collaboration between diagnostic devices and the professional experience of radiologists, AI intervention seems much easier than in other fields, but this is often not the case. Many times, the patients orient themselves according to the doctor, which is not applicable in the case of AI. Another limitation rests in the controversies regarding medico-legal liability. In the field of radio-imaging in Romania, the implementation of AI systems in diagnosis is at its beginning. An important aspect of this is raising awareness among the population about these assistive AI systems and, also, awareness of the technological evolution of AI among medical staff. This narrative review manuscript analyzes the existing literature data regarding the medico-legal aspects of AI application in radiology, highlighting the controversial aspects and the lack of statutory legislative regulations in Romania. **Methods**: A detailed search was conducted across three electronic databases including MEDLINE/PubMed, Scopus, and Web of Science, with 53 papers serving as the literature corpus of our review. **Results**: General requirements for artificial intelligence systems used in radiology have been established. In the radiological diagnostic process, there are five levels of AI system implication. Until now, completely autonomous AI systems have not been used. Regarding liability in the case of malpractice, at the currently accepted legislative level, most of the time, the radiologist is liable for their own fault or non-compliant use of diagnostic AI systems. Controversies arise in the case of radio-imaging diagnosis in which AI systems act autonomously. **Conclusions**: In order for AI diagnostic radio-imaging systems to be implemented, they must meet certain quality standards and be approved. The radiologist must know these systems, accept them, know their limits, and validate them in accordance with their degree of involvement in radiological diagnosis. Considering the evolution of technology in the Romanian medical system, including radiology, in the future, an alignment with the legal standards established/proposed at the European level is desired.

## 1. Introduction

Artificial intelligence (AI) represents the simulation of human intelligence processes by machines or computer systems [1]. It is gaining an increasing amount of influence in various fields of activity, including the medical field.

The use of AI appears to be more difficult in the medical field due to human involvement in this activity, for example, the relationship developed between the patient and the doctor. However, it is evolving even in this field.

In radiology, where diagnoses are based on collaboration between diagnostic devices and the professional experience of radiologists, AI intervention seems much easier than in other fields, but this is often not the case. Many times, the patient relies on advice from the doctor, which is not applicable in the case of AI. Another limitation is represented by the lack of medico-legal liability regarding AI [2]. This narrative review article aims to debate AI’s impact in radiology among medical personnel and patients, with a particular focus on the Romanian medical and legal context.

## 2. Materials and Methods

A detailed search was conducted across three electronic databases including MEDLINE/PubMed, Scopus, and Web of Science, using combinations of the keywords ‘artificial intelligence’ and ‘legal implications’; or ‘artificial intelligence in radiology’ and ‘legal implications’; or ‘artificial intelligence’ and ‘malpractice’; or ‘artificial intelligence’ and ‘legal liability’; or ‘artificial intelligence’ and ‘legal responsibility’; or ‘artificial intelligence’ and ‘legal regulation’; or ‘artificial intelligence’ and ‘legal implications in Romania’. Articles published in English up until November 2024 were considered. A total of 490 published papers in the English language were initially analyzed. Two independent reviewers screened the abstracts and assessed each study’s relevance, based on the inclusion and exclusion criteria.

The following inclusion criteria were established: abstract explicitly highlights the topic of AI in radiology and its legal implications—this criterion helped us to only choose research papers having both components and to remove the papers talking about AI in some other context; the paper’s focus aligns with the chosen research focus—while going through the paper, if it did not cohesively talk about AI in radiology and its legal implications, then that specific paper was not included in our analysis; abstract and keywords contain key terms related to the topic; open access papers.

The following exclusion criteria were established: duplicate papers; non-English papers; content focuses only on a specific niche sub-field of research regarding AI; abstract does not cover AI in radiology and its legal implications; full article not accessible—papers that seemed relevant from the title and first information, but were not accessible, were excluded from this analysis.

In the end, we included 53 papers, which served as the literature corpus of our review. A total of 65% of them were published in the past 5 years. However, considering the aim of this paper, international and Romanian legislative acts, and laws, were analyzed for the discussion, resulting in the complete reference list being wider than the included articles.

## 3. Findings

### 3.1. Involvement of AI in Medicine and Radio-Imaging Diagnosis—General Considerations

In our society, the rate of AI involvement in various fields is around 86% [3].

AI is also implemented in radiology to optimize interpretation in areas where the success rate is low. For example, in mammography reports, where the radiologist error rate is high (between 10% and 30% of cancer cases), AI is considered an alternative diagnostic tool [4].

Artificial intelligence is a form of human-made computer programming that uses human skills. It is based on machine learning and deep learning [5].

Taking into account the evolution of the mammography technique, and the adoption of digital mammography (starting in the 2000s), computerized systems have been increasingly favored for diagnostic assistance [6,7,8].

The US (United States) Food and Drug Administration (FDA) first approved computer-aided detection (CADe) for clinical use in 1998. It became widely used, and in 2002, it was also approved by the Centers for Medicare and Medicaid Services (CMS) [9,10].

In the beginning, CADe worked as a double-reading system in which mammographic changes were underlined by a computer, which was followed by checking and validation by a radiologist [10].

This double-reading option is widely used in Europe, and since 2016, CADe has been used in 92% of mammographic screenings in the US [9].

There are studies reporting improvements in the treatment of small invasive cancers due to the use of CADe reports in mammography [10].

Other studies have stated that radiologist reporting times are increased due to CADe, but that its benefits are considerable [11].

There are also studies that state that the use of CADe does not increase diagnostic efficiency [12,13].

A new step in breast cancer diagnosis is represented by the implementation of tomosynthesis, which provides 3D images of the breast, increasing diagnostic effectiveness [14].

Considering the different methods of mammographic diagnosis, from the point of view of the protocol used (device-dependent or technician-dependent), distinctions should be made in the application of AI, and it should be adapted to the quality of mammography [15].

In the beginning, AI was mainly applied in mammography in triage for easier cases so that radiologists could dedicate enough time to difficult cases [16].

However, there are also studies that support the full involvement of AI in mammographic diagnosis. Approximately 37 million mammograms per year in the US are analyzed via AI and thus eliminated from radiologist workloads [4].

Even if a medical conclusion can be reached by applying AI to interpret mammograms, from a legal point of view, this subject is controversial. In order to practice, a radiologist needs a medical license for free practice, as they are under their country’s medico-legal jurisdiction. It would be unusual for such a license to be assigned to AI. In China, a solution was reached whereby AI was awarded a “medical license” [17,18]. However, this situation is uncommon.

There are some countries in which double reading is used in mammography, to reduce the incidence of misdiagnoses. One study concluded that 16–31% of detectable cancers are missed when screening mammograms are assessed by a single reader. Therefore, the use of CADe is preferred instead of a second radiologist’s mammogram report [11,19].

The development of AI in the medical field makes direct diagnostic applications possible. Studies have been performed on mammogram databases from the UK (United Kingdom) and the US using AI diagnosis, and the rate of false positives was reduced by 1.2% in the UK and 5.6% in the US; for false negatives, the rates were reduced by 2.7% and 9.4%, respectively.

Transferability is also a very important aspect to consider. An AI radiological diagnostic tool implemented and tested in one country should be applicable in other geographical regions. This is very important for the application of AI for diagnosis in disadvantaged regions [20].

The founders of AI programs must permanently improve their systems and subject them to permanent checks and approval from local authorities who put patient safety first.

There is also a wide range of AI and machine learning applications in cardiovascular imaging with reference to image acquisition, processing, automatic measurements, quality evaluation, matching new images with previous ones, diagnostic guidance, and even diagnosis [21,22,23,24].

### 3.2. What Does Med-Mal Mean? AI Implications in Radiological Fields

According to published data, radiologists were generally optimistic about the incorporation of AI in medical imaging practice. However, low levels of AI education and knowledge remain a critical barrier. Furthermore, equipment errors, cost, data security, operational difficulties, ethical constraints, job displacement concerns, and insufficient implementation efforts are integration challenges that should merit the attention of stakeholders [25]. The integration of AI in radiology raises significant legal questions about responsibility for errors [26].

Med-mal represents a specific part of tort law that protects and compensates individuals harmed during medical practice [27].

Tort means “a civil wrong, other than breach of contract, for which a remedy may be obtained, usually in the form of damages” [28].

In medical practice, a radiologist cannot be considered guilty if their activity falls within the average level of training, and they did not intentionally or negligently cause harm to a patient [29].

When a doctor causes harm to a patient, fault liability requires that the act be proven to be intentional or negligent [30].

For a plaintiff to have a medical malpractice claim, four conditions must be proven: duty, breach, causation, and loss [31].

In order for a patient to claim med-mal, a doctor must cause them harm through their direct actions.

Regarding AI liability, this occurs when the manufacturer has breached/neglected medical standards in the field of application [32].

It must be taken into account that different societies and countries have different medical standards [33].

Most of the time, AI applications are above known standards [17].

Regarding medico-legal liability, in addition to the attending physician, the hospital and the medical superior are considered defendants. In this legal system, the hospital is considered directly responsible for the physician’s negligent acts. The hospital is responsible for hiring competent employees and for ensuring their adequate training and professional conditions [5].

There are also situations where an on-call doctor is independent of the hospital, having limited activity in this service. In these situations, the hospital cannot be held liable for this doctor. However, if this situation is not known to the patient, they can take the hospital to court [5].

The manufacturers of some medical devices may be directly responsible for damage caused by the use of these products if they cause harm to a patient due to defects for which the manufacturer is responsible. Many of these products have usage authorizations from higher forums in the field. Under these conditions, the manufacturer is not directly liable. However, in the application of AI in radiology, this theory is not generally valid [31]. From this perspective, it is considered that the final decision rests with the radiologist, who must validate the result obtained by using AI [5].

### 3.3. AI and the Legal Aspects of Malpractice

Due to the need to streamline workloads, and to ensure quality service in the face of a growing need for radio-imaging investigations, the introduction of AI as an adjuvant or even for diagnostic purposes is becoming a fact. This situation also results in the need to establish clear and concise conditions for malpractice law.

Since 2019, there have been over 300 AI applications for which FDA review and approval have been requested in the United States.

In January 2023, there were approximately 520 such applications registered with the FDA; of these, 396 were in the field of radiology [34].

Within the current legal status, it is difficult in cases of malpractice to establish the person responsible for radio-imaging misdiagnosis when using AI. Usually, a person who can anticipate and prevent the occurrence of any kind of harm to a patient and does not do it is the one responsible in this situation [3].

There are several aspects which make it difficult to assign legal responsibility. For example, it is difficult to assign legal responsibility in situations in which a diagnosis requires the joint action of several users. Whenever AI is used in diagnosis, it is challenging to assess the level of violation of accepted standards. Due to these controversial aspects, it is therefore difficult to provide clear standards in court, through which responsibility could be further assigned [5].

Due to a lack of legal support, a lack of precedents regarding the application of AI in radio-imaging diagnosis, and the errors that may occur in the situation of medical malpractice, assigning responsibility is challenging and often inconclusive. In order to introduce this aspect into the medico-legal circuit, it is necessary to refer to traditional legislation. Through their current fast evolution, AI systems are becoming increasingly autonomous, so it is inappropriate and incorrect to attribute responsibility to human beings, who are associated in various ways with these systems as producers, implementers, or users [35].

The legal system is based on liability for intentional harmful acts, and it analyzes the effects of intention—causes. We refer to intention particularly in situations which involve human beings, without being able to assess whether intention can be found by analyzing the harmful behavior of some AI systems, regardless of the field. Upon integrating AI into the medico-legal liability system, the idea of intention–cause becomes controversial [36].

In carrying out the medical acts of diagnosis, management, and treatment of a patient, several individuals, from medical and administrative staff to even those who produce and implement AI in the medical system, are involved. Thus, it is difficult to assign medico-legal responsibility. When a plaintiff acts in court against everyone, it is considered excessive and expensive [5].

### 3.4. Does AI Medical Error Lead to Physician Liability?

A doctor may be strictly liable for their direct actions in diagnosing and treating a patient. This also applies in radio-imaging interpretation. Controversy occurs when AI is involved in determining radio-imaging results.

For example, several individuals are involved in obtaining a mammogram: the doctor who recommended it, the technician who performed the procedure, and the radiologist who interpreted the images. In cases where AI is involved in interpreting images and formulating results, others could also be considered for the assignment of liability in the event of medical malpractice: the producer of the AI program, the person who implemented it, the person who purchased it, the person who missed the diagnosis, the radiologist (even if they have no direct role in formulating the final imaging report), etc. However, none of these situations are rational when assigning medico-legal liability.

However, in situations where double radio-imaging interpretation is practiced (radiologist and AI), in the event of malpractice, even if the AI interpretation is superior to the human one, the attribution of responsibility to the radiologist comes into question [5].

### 3.5. Does AI Medical Error Lead to Hospital Liability?

Although not directly responsible for an AI diagnostic error, a hospital could be considered partially responsible for choosing an inappropriate and inadequate AI system.

Even though, in many sectors of activity, as well as in many countries, the application of AI in medicine is at its beginning and it is difficult to opt for a specific AI system, before introducing an AI system into current practice, various testing methods can and should be applied.

However, unilaterally assigning responsibility to the hospital and the administrative service would engender reluctance in the implementation and subsequent support of AI in the medical field. This is a precedent for other health services as well [36,37].

Thus, until there is legal and medico-legal support for the implementation of novel AI in medicine and radio-diagnostics, hospitals can choose to maintain and develop radiology with human resources or, in the case of larger hospitals, to implement and use previously tested AI systems. In the latter case, in the event of a malpractice dispute, the hospital could be assigned some liability and could be required to be present in court.

The application of AI in medicine and in various sectors of activity, including radio-imaging diagnosis, is a controversial topic, especially because of uncertainty in med-mal situations [5].

### 3.6. Does AI Medical Error Lead to Manufacturer Liability?

Most of the time, when a medical conflict involving error in an AI system occurs, the manufacturer/programmer is among the first to be held liable. They are probably among the few people who could technically explain an error. The problem that arises in this situation refers to the impossibility of disclosing a manufacturing and implementation secret or patent in court, with reference to intellectual property rights and product security.

On the other hand, the technical explanations of some programs and the descriptions of some mathematical algorithms are considered over-specialized, as they are not fully understood by the majority present in court [36,38,39].

In addition, a diagnostic error caused by AI through a technical/construction defect does not represent a real malpractice conflict. This “manufacturing defect” error falls under other legislative regulations. Moreover, any product introduced into the market must meet certain standards, and be approved by an authorized structure.

There are cases where liability can be attributed to the manufacturer of an AI system if a physical defect causes a direct injury to a patient, although this is rarely applicable in radio-imaging diagnoses [5].

### 3.7. AI-Related Malpractice Causes and Legal Processes

Current AI technologies modify the acknowledged standards which are considered in medico-legal cases. Even with their high performance, it cannot be said that diagnoses are certain, so medical incidents are inevitable [31].

With the evolution of techniques and the large-scale application of AI in diagnosis, it is natural to change what is considered as the accepted standard. It is thought that approximately 17 years will pass from the initiation of a new AI practice to the moment in which, through evolution, it will help in redefining the accepted standard in a given field [40].

In the case of a med-mal trial involving a diagnostic error of AI, it is difficult to find an outside witness who could support the AI clause with certainty. One solution would be to compare the AI’s diagnostic history in similar situations to that of the plaintiff [41].

In a situation where, in all previous cases, the imaging diagnosis of AI is compliant, it is considered that, in the current case, the expected standards are met and an expert witness is no longer necessary. In these cases, the AI system can be considered both a defendant and an expert witness, which is accepted in court if knowledge, training, experience, and education allow it [42].

Another means of establishing a diagnostic standard is by testing the same patient images using multiple AI systems. Shortcomings occur in situations/areas where AI involvement in medicine is at its early stages, and there are no alternative AI systems. This method is a controversial one because it is difficult to accept the evolution of AI techniques in the diagnostic sphere, as they can even exceed human performance [43].

The transition from a human-based medical diagnostic standard to one based on AI technology can be difficult for both the community and courts to understand and accept [44].

One option to consider is calling expert AI programmers as witnesses who can comment on a system’s “reasoning errors”. However, their opinions cannot be proven as decision makers in court often find it difficult to understand them due to the technical terms involved. In addition, human radiology experts can be considered as witnesses who can analyze the imaging diagnoses of an AI system, in which case there would be a contradiction regarding the use of AI to the detriment of human resources for diagnosis in some imaging sectors. This is because the application of AI in radio-imaging diagnosis is gaining ground in some specialties due to its superior diagnostic ability.

In small communities that are subject to local legislative regulations, it is difficult to apply and use AI as a diagnostic method.

Considering the absence of clear and concise legal regulations regarding med-mal cases, providers of AI diagnostic systems could choose between refusing their implementation until the legal framework is clarified, or applying them under existing conditions with possible uncertain legal consequences [5].

In the case of first option, this represents a slowdown in technological evolution and in the applicability of AI in medical imaging diagnostic systems.

### 3.8. Ethical and Fairness Aspects Regarding the Use of AI in Radio-Diagnostics

For the appropriate use of AI systems in diagnosis, it is essential to respect ethical principles, as well as fairness in the application of these systems.

Ethics means the “application of values and moral principles to human activities... and seeks to find reasoned, consistent, and defensible solutions to moral problems” [45].

Standard medical practice requires compliance with ethical principles.

These ethical principles do not have absolute value; they are generally valid, with adaptations related to the specifics of the region in which they are applied, variations in the population, and the specifics of the area and principles already existing in certain regions to ensure equal access to the medical system.

In agreement with this, considering that in Romania the application of AI in radio diagnostics is at its beginning, good practice in this field involves aligning with those absolute values related to general ethical principles (further discussed). Likewise, in our country too, these generally valid principles will be adapted depending on the regional variation of the population and the specifics of the area in question.

The oldest and most important principle refers to respect for people and for justice. In this direction, in the fourth century BC, physician and philosopher Hippocrates beseeched physicians to “do no harm” [46].

The first edition of *Principles of Biomedical Ethics* was published in 1979, by Tom Beauchamp and James Childress [47]. Additionally, the same year, the *Belmont Report* published the guidelines for the responsible use of human subjects and human subjects’ data in studies [48].

So, Beauchamp and Childress established four ethical core principles of autonomy, beneficence, non-maleficence, and justice [49]. In addition to these, a fifth principle was added—explicability [50].

Autonomy in healthcare means the respect for a patient’s decision to adhere to diagnosis and treatment. The use of AI systems could interfere with autonomy values like respect for privacy, human dignity, and transparency. Only morally endowed elements can act in a certain way. It is hard to imagine attributing moral status to an AI system. In most situations, to comply with these fundamental principles concerning respect for the patient, AI systems are supervised by humans and do not act autonomously. In cases of diagnosis using AI systems, autonomy suggests that patients have to be informed about AI’s use in the diagnostic process, and subsequently give their consent [51].

The principles of non-maleficence and beneficence are interconnected. Any medical action for the benefit of the patient must first of all respect the Hippocratic injunction “primum non nocere”. Any medical action decided must also anticipate negative effects, and be carried out in accordance with the principle of minimizing any risk of harm [51].

An example from the radiological practice, mammography, is the adaptation of AI systems so that, through a protocol, it ensures radiation doses are as low as possible, without compromising diagnostic quality [52].

Beneficence is the moral principle of undertaking a medical act with the aim of improving the patient’s health; in the case of radio-imaging, through a correct diagnosis. While non-maleficence means avoiding harm, beneficence means a positive action. Through using AI in mammography, beneficence can mean, for example, the improvement of AI screening recommendations or imaging diagnosis accuracy, for a prompt and effective therapy [51].

The principle of justice in healthcare signifies appropriate care (diagnosis, treatment) for patients, and the fair and equitable distribution of care, without social biases or inequalities.

In this case, the development of AI algorithms needs to respect the justice principle, otherwise inadvertent errors may occur.

In breast cancer screening, there is a wide variation in diagnostic accuracy depending on geography, racial/ethnic background, access to new technology (tomosynthesis), and socioeconomic status. Under these circumstances, machine learning algorithms can lead to diagnostic errors [51].

In using AI in healthcare, the principle of explicability also appears. It comprises intelligibility and accountability. Intelligibility means the ability to understand how AI systems work, and accountability means clarifying who is responsible for AI’s actions [50].

Intelligibility may be referred to as a “black box problem” because of difficulties in understanding AI algorithms. However, the confidence which radiologists could gain in AI systems depends on this understanding.

Accountability means to assign responsibility. This principle is very important in the trust placed in, and the safety of the use and application of, AI systems and in removing reluctance related to technological evolution. Increased attention is mainly due to the development of AI systems that aim for autonomy in diagnostic activity [51].

The table (Table 1) below summarizes the five ethical principles involved in the use of AI systems detailed previously.

The ethical application of AI in radio diagnostics must take into account the five general ethical principles for AI use in healthcare. For AI use in radiology, there is a consensus among the American College of Radiology (ACR), the European Society of Radiology (ESR), the Radiological Society of North America (RSNA), the Society for Imaging Informatics in Medicine, the European Society of Medical Imaging Informatics, the Canadian Association of Radiologists, and the American Association of Physicists in Medicine.

AI systems with applications in medicine are expanding and are tending to become highly autonomous, which also entails the risk of error and even ethical and social consequences.

The use of AI under ethical conditions refers to a kind of use in which benefits are sought with the minimization of negative effects, with respect for human/patient rights, with respect for the right to privacy and security, and with the fair distribution of responsibility in case of any damage [53].

Data ethics in the use of AI in radiology refer to trust in the acquisition of data, their management, and their processing. All of this must be done with informed consent from the patient while respecting data security and protection, transparency, objectivity, and fair access to these data [54].

Transparent communication with the patient is necessary, so that they understand the purpose, risks, and benefits of storing their own data [55].

The ethics of algorithms and trained models also come into the discussion. Although human beings decide based on rational opinions, knowledge, values, and beliefs, AI forms opinions based on preset data, algorithms, and action models. To transmit the human model of decision-making, which also considers equality and fairness, to AI, human values should be “transferred” to the AI system [56].

Humans/programmers/those who implement AI systems should anticipate and prevent any inappropriate and unethical use of AI [57].

Data storage and transmission data protection are also very important concerns. AI systems implemented in imaging diagnosis must be protected from external attacks for malicious purposes [58,59,60,61], using robust security measures like encryption and anonymization techniques, strict access controls, and techniques for tracking data usage.

There are strictly established privacy regulations, which must be accepted by the developers and users of AI systems (Health Insurance Portability and Accountability Act—HIPAA in the United States and the General Data Protection Regulation—GDPR in the European Union). In this way, trust in AI systems is ensured, patient autonomy is maintained, and statutory ethical and legal standards are adhered to [62].

When applying AI systems to radio-imaging diagnostics, the ethical principles of practice must be considered. AI is a complex system that relies on advanced technology and mathematics, but conscious ethical values and actions that take moral aspects and non-harm to human beings into account are questionable. Even unintentionally, some automated actions of AI can cause discomfort to patients, society, and even the manufacturer [53,63].

The application of AI systems in radiology must also respect the concept of fairness, which refers to the development and application of equitable AI systems that can ensure access to appropriate diagnosis and treatment for all people, without discrimination [62].

The biases identified in the use of AI in radiology refer to data bias (coming from data used for algorithm development), algorithmic bias (from the learning mechanism of the algorithm), as well as biases resulting from human interactions: from AI–clinician interactions and from AI–patient interactions.

A very important topic is that of developing strategies to combat these biases [64,65]. Data biases come from the collection and organization of data used for AI algorithm development. These are classified as minority bias, missing data bias, informativeness bias, and the mismatch of “training-serving” [65].

Minority bias refers to the situation in which the initial data group used to implement an AI system or an adequate learning process is insufficient or uneven. Errors may occur if the AI system is applied to an underrepresented group.

Missing data bias happens when there are no randomly missing data from some groups and, therefore, the AI system has a prediction shortage.

Informativeness bias occurs when the essential elements used by AI for identification are less specific in some groups.

Mismatch of “training-serving” means inconsistency between the data used in AI training and those used in AI application. This can occur in a situation where selection criteria for data/information used for an AI system’s development are inappropriate, or when the application of AI is carried out on a particular group excluded from the initial AI “learning process” [62].

Algorithmic biases are deficiencies resulting from the development and implementation of AI systems. These are label and cohort biases [65]. The label bias occurs when the AI system views particular elements as universally valid. Cohort bias appears when AI systems are created using the usual groups, ignoring particular cases.

Some other important biases come from the interaction between AI systems and clinicians, and between AI systems and patients.

Considering the interaction between AI systems and clinicians/radiologists, one of the situations that leads to error is related to absolute trust in the advice of an AI system [62]. In this regard, there is a study that proves that inadequate advice from AI systems in interpreting mammograms negatively affects the mammogram interpretation performance of professional radiologists, not just beginners [66].

Another interaction bias comes from accepting incorrect AI system recommendations, so that through this acceptance the AI learning system is damaged, which will perpetuate the error, thus considering it correct further down the line.

In the case of excessive alerts from the diagnostic AI system, the radiologist will develop a resistance to these alerts, eventually ignoring even correct alerts—hence another element of interaction bias [62].

From interactions between AI systems and patients, result biases like privilege bias, informed mistrust, and agency bias may occur [65].

Privilege bias refers to situations in which there is no access to AI systems for all the patients who need it.

Informed mistrust means the skeptical attitude of patients towards the application of AI systems, generally due to known and perpetuated inequities.

Agency bias occurs when there are protected groups that do not participate in the “learning” and development processes of AI systems [62].

The existence of these biases in the application of AI has led to the development of strategies to combat them.

An important role in this regard is played by the appropriate selection of the data used for the development of AI systems, using a wide range of selection, and taking into consideration demographic needs and health systems, the particularities of the target population, socioeconomic and cultural status, the existence of different stages of the disease, and different age groups. This attitude assures greater diagnostic accuracy, greater addressability of patient groups, and the increased performance of AI systems [67].

Algorithmic biases can be identified and corrected through the regular control and validation of the AI system’s functioning. These periodic checks for the correct and adequate operation of AI diagnostic systems are also necessary due to the dynamic character of the medical field, which is in a state of permanent evolution [62,68]. Under these conditions, it becomes necessary to create departments that ensure this periodic control, at a hospital level, which can also apply measurements to constantly improve the AI systems’ algorithmic levels, as to ensure correct and fair diagnostic application [62].

To combat biases that arise from the interaction of clinicians and patients with AI systems, adequate and continuous education and information is necessary regarding the involvement of AI in medicine and diagnosis; at the same time, the acceptance of technological evolution is a must. Knowing the weaknesses of AI systems protects clinicians from total trust and possible medical error.

Likewise, clinicians can actively participate in improving AI systems through their previous experiences with known AI systems.

Informing the patient about AI systems is very important, as is recognizing their biases. This is possible through media coverage, interdisciplinary collaboration, and exemplification with previous experiences in the field.

In this way, reluctance towards technological evolution is removed and openness towards, and even indirect involvement in, the development of AI systems is also possible [62].

**Figure 1:**
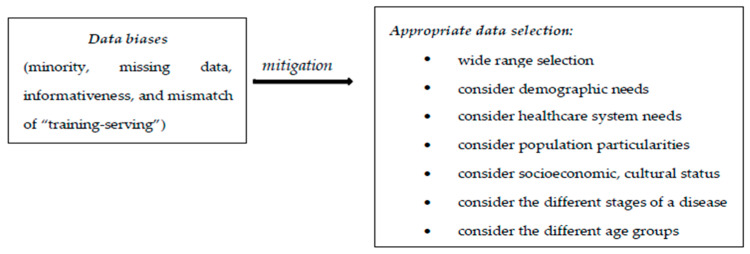
Data biases.

**Figure 2:**
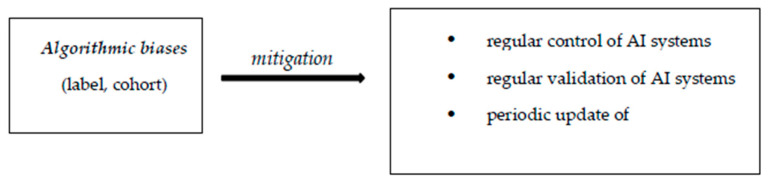
Algorithmic biases.

**Figure 3:**
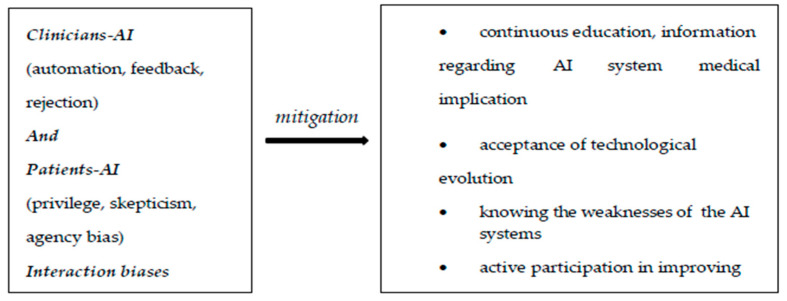
Interaction biases.

In the figures (Figure 1, Figure 2 and Figure 3) below we have represented the types of biases discussed and some ways to prevent them [62].

## 4. Applying AI in Radiology—Current Legal Regulations: What About Romanian Practice?

At the European Union (EU) level, the AI Act was developed, and it considers a series of legal regulations regarding the use of AI in radiology.

Although these medico-legal aspects involving the use of AI in diagnosis have been debated for a long time, and a series of hypotheses have been issued, definitive legal regulations regarding this aspect have not yet been established [69].

The development of the EU AI Act [70] is based on high safety requirements in the application of AI in radiology, as well as in the prevention of harm and negative effects on users [71].

### 4.1. General Requirements for AI

AI systems must be technically adequate so as to prevent any harm or any external involvement with harmful intentions (radiological images in deep learning systems can be attacked online and compromised, resulting in diagnostic errors) and the use of AI must comply with current privacy and data protection requirements (General Data Protection Regulation (GDPR)) [72,73,74].

Another general condition for AI systems is transparency. This refers to the possibility of an AI system to be explained to a patient in understandable non-technical terms so that they understand the advantages and limitations of the AI’s diagnostics system [72,75].

“Diversity, non-discrimination, and fairness” are other general conditions for AI applications [71]. In addition, the use of AI systems involves preserving well-being in society and the environment [72].

### 4.2. About the European Union AI Act

The European Union AI Act (EU AI Act) is based on risk assessment, GDPR, and the legislative framework for medical devices [71].

AI systems in radiology intervene in prevention, diagnosis, follow-up, prognosis, and even treatment; thus, they are classified as medical devices [76,77]. They must have CE certification (National Certification Center for Medical Devices) and are assigned a degree of risk [77]. Many AI systems used in radiology are classified as high-risk, as they are medical devices that can interfere with a patient’s physical condition and evolution, potentially causing harm [71].

An essential element specified in the AI Act is that AI systems must be carefully and constantly supervised by human resources [72,78].

Currently, the EU AI Act does not accept the autonomous activity of AI systems used in medicine. They must operate under close and permanent human supervision, from the implementation and training phase to the verification and correction of results [72,78,79].

In the radiological diagnostic process, there are five levels of implication of AI systems [80,81], which are presented in Table 2.

Until now, completely autonomous AI systems have not been used in radiology. For that reason, the EU AI Act supports the principle of oversight in the use of AI systems, so that high-risk medical systems are constantly supervised by humans. Non-compliance with this condition is an illegal action. Human oversight in radiology translates into the permanent involvement of radiologists and active actions to control and guide the diagnostic activity of AI systems, as to achieve a qualitative and safe result, in accordance with legislative obligations and requirements. Radiologists’ oversight is required at all levels of AI automation, except level 4, the total automation level [71].

The most common AI systems for radio-imaging diagnostics are CAD-type, which perform automatic lesion detection, that must be validated later by the radiologist. They work across the entire range of radio-imaging diagnostic methods.

Level 1 and level 2 AI radio imaging-assisted diagnosis is frequently encountered in mammography interpretation as an additional tool in the double-reading process, but also in the selection of normal images.

This multidimensional radiologist–AI diagnostic approach and the degree of AI involvement can change the human diagnostic attitude, which can ultimately generate errors. This was analyzed in a stroke diagnostic study at a German university hospital [83].

### 4.3. Concerning Medico-Legal Liability in the Combined Activity of a Radiologist and AI

Civil liability experts, together with the European Parliament, have shown the need to revise civil liability legislation [84]. In this regard, reference is made to non-contractual civil liability in the case of the use of AI systems, with the aim of facilitating information and providing evidence in malpractice situations involving AI. Reference is also made to a directive on liability in the event of damage caused by defective products [85].

These legislative projects work together with the EU AI Act to enforce liability legislation in cases of malpractice involving AI systems [71].

In order for a radiologist to be liable for their actions, they must have caused harm to a patient, there must be a clear causal relation between the radiologist’s activity and the harm caused, and there must be fault [71,86]. This results in liability for harm without fault on administrative grounds and civil liability in relation to defects in medical equipment, as well as liability based on fault (non-contractual civil liability and criminal liability) [71,85,86,87]. Non-contractual civil liability refers to an action or omission on the part of the radiologist that, due to fault or negligence, causes harm to a patient. Non-contractual civil liability refers to an action or omission on the part of the radiologist that, due to fault or negligence, causes harm to a patient. However, criminal liability refers to a committed action on the part of the radiologist, causing harm, which is subject to the criminal code [71].

Regarding liability in the case of malpractice, at the current accepted legislative level, most of the time, radiologists are liable for their own faults or non-compliant use of AI diagnostic systems [71] (Table 3).

There are situations in which the responsibility lies with the hospital’s radiology department if erroneous information is provided to the radiologist regarding the use of AI and, hence, harm is caused [88].

If it is proven that an AI system does not comply with certain manufacturing standards and is defective, causing harm to a patient, the responsibility rests with the manufacturer [85].

There are increasing questions regarding the attribution of medico-legal liability to autonomous AI systems [89]. However, at the current level, the European Union’s legislative system does not encourage this [90], and there is no evidence of fully automated AI systems being used in radiology [71].

AI systems in radiology can interfere with radiologists’ work to varying degrees, potentially influencing their degree of liability in the event of a medico-legal conflict [71,91].

In situations where an AI system is issued as a support tool, the radiologist is the one who interprets and has the final decision on the result, as well as the medico-legal liability in case of harm [92].

If AI behaves as a tool to assist a radiologist’s activity, it acts independently in pre-established and directed situations, and the validation and final decision regarding the imaging result lies with the radiologist. In these situations, the responsibility rests with the radiologist, with the exception of cases where a defect in the product is proven—cases in which the responsibility lies with the developer/manufacturer [71].

In cases of radio-imaging diagnosis in which AI systems act autonomously, they cannot be directly assigned medico-legal responsibility because they do not have a legal personality [71]. Controversies arise in the latter situation. It is difficult to attribute legal personality to a medical device, and it is also difficult to attribute responsibility to other humans. Although there are actions being taken in this direction, the EU legal system does not provide support for the time being. Diagnostic errors can arise from the combined activity of radiologists and AI systems either through erroneous prediction by the AI or through a radiologist’s verification deficiency. Hence, different variations in liability arise in the event of a medico-legal incident [71].

Errors of commission refer to a situation in which the commendation of an AI tool is valid, but the radiologist ignores it, resulting in an erroneous result. In this situation, the responsibility lies with the radiologist [93,94,95].

An error of omission refers to a situation in which an AI system makes an erroneous diagnostic recommendation, and the radiologist does not verify it but only validates it. In this situation, the responsibility lies with the radiologist [93,96].

If an AI tool provides an erroneous/false negative diagnostic recommendation (normal instead of a true pathological result) and the radiologist does not verify the result but rather validates it descriptively with normal elements, an error of omission action occurs. In this situation, there is a high risk of a legal and even criminal response for the radiologist [97].

For the beneficial application of AI systems in radiological diagnosis, they must act with maximum efficiency and minimal risks, which requires the combined action of radiologists, engineers, mathematicians, and lawyers. Through this interdisciplinary collaboration, errors and biases can be minimized, the operating algorithms of AI systems can be understood and further used appropriately, and the medico-legal framework can be better evaluated with the conscious acceptance of uncertain situations.

Radiologists must also accept the help of AI systems and even welcome this support [57].

## 5. Discussion and Conclusions

In the field of radio-imaging in Romania, the implementation of AI systems in diagnosis is at its beginning.

Radiological diagnosis is either traditional (level 0) and performed exclusively by the radiologist, who responds in the case of a medico-legal conflict, or it is assisted through first-generation computer-assisted diagnosis (CAD). In each of these situations, the medico-legal responsibility lies with the radiologist, and no actual involvement of AI in the diagnosis can be assessed.

Considering the evolution of technology in the Romanian medical system, including radiological technology, in the future, an alignment with the legal standards established/proposed at the European level is desired. Initially, specific medico-legal elements should be integrated into the local legal system.

At this moment, there is no dedicated legal framework for the application of AI systems in Romania. However, various already existing regulations may be applied. Regarding data protection, the GDPR could also be used in terms of data used by AI systems. Another regulation which could be applied is Law 190/2018, regarding the processing of certain types of personal data, the role of data protection officers and certification bodies, and the applicable sanctions for public and private entities.

The integration of the EU AI Act is expected in Romania, where the EU Commission proposed the Artificial Intelligence Liability Directive (AILD). This directive establishes common legal ground regarding non-contractual civil liability for harm produced by the use of AI systems.

To establish a high common standard for network and data security across EU member states, it was requested to integrate the NIS Directive. This is a directive regarding measures of high-level cyber security across the EU. On 9 January 2019, Romania promulgated Law 362/2018. This law appoints CERT-RO as the national authority responsible for network and data systems security. It has also been designated as the main interface for cooperation within the authorities of EU member states.

In January 2023, NIS Directive was replaced by NIS2 (EU Directive 2022/2555) [98]. EU member states had a deadline of September 2024 for introducing these directives into their national legislation frameworks. This last directive appeared as a correction to the previous one, and an adjustment to high-tech progress.

On 12 March 2024, the European Parliament adopted the Cyber Resilience Act, which refers to cyber-security assessments and requirements for digital products, automatic security updates, and the obligation to report vulnerabilities and incidents to ENISA (EU agency dedicated to enhancing cyber-security in Europe). This will be followed by the formal adoption and transformation of this act into law for EU member states to follow.

In Romania, there is governmental interest in applying AI in various areas of activity. With this purpose in mind, The Romanian Committee for Artificial Intelligence was established by the Minister of Research, and the Scientific and Ethical Council in Artificial Intelligence came into being. Also, a Coalition for AI has been proposed for development purposes and for unified opinions towards officials.

Increased attention is being paid to the implementation of draft law PL-X No 471/2023, which targets the responsible use of technology to combat the deep-fake phenomenon (combating misinformation and preserving the messages’ integrity).

The Romanian government’s interest in implementing AI systems is also reflected in the development of the National Strategy for Artificial Intelligence, which promotes transparency and accountability in AI systems.

Therefore, as an EU member state, Romania will have to implement the regulations of the EU AI Act into its own laws. In this regard, Romania has joined the EU Commission’s White Paper on AI.

Several important perspectives regarding Romanian law showcase the fact that in Romania there is still no clear definition of AI systems and, at the same time, AI systems are not granted legal personality.

In order to align Romanian law with the EU AI Act regulations, it is necessary to implement a medico-legal framework that complies with the Act’s directives, as well as specific elements adapted to the local law. This demands the establishment of control bodies, in order to ensure compliance and to reinforce those regulations.

Until now, in Romania, there has been no acknowledged legal assessment involving AI systems. This may be due to the lack of a legal framework, but also because AI systems are still in their early stages, although manifesting a rapidly increasing development. Another explanation could be found in the law court’s reluctance to involve AI systems in the process, due to controversial legal circumstances.

The Romanian Ministry of Research, Innovation and Digitalization, and the Authority for the Digitalization of Romania (ADR) have the aim to develop and implement the regulatory and operational framework for AI. This is how the National Strategy for Artificial Intelligence was developed, which ensures that the following principles are acknowledged in the implementation, application, and development of AI systems: “respect for human rights and democratic values; holding AI under the control of human intelligence and action as the final actor in decision-making; respect for diversity and equality among users and gender equality, in order to give access to AI products and services to anyone; security and safety with regard to the services offered and the data processed in case of risk or threats of cyber-attacks; and transparency and trust on the operation of AI services” [99].

In addition to the undergoing implementation and development of the medico-legal framework in Romania, the main challenges in implementing AI systems detailed above are also very important and mainly refer to ethical issues, the “black box” dilemma, personnel training, standardization, and limited datasets [100].

An important concern is to raise awareness among the population about these AI assistive systems and, last but not least, to raise awareness about the technological evolution of AI among medical staff. Reluctance to use AI systems as a diagnostic tool also results from ignorance towards legal aspects, as well as from their controversial nature.

There is a study that recommends an assessment of the controversial aspects of the relationship between technology and society, made by David Collingridge (English philosopher) in 1980, in *The Social Control of Technology* [101]. In this study, it is mentioned that when there is technological evolution, the effects on society are difficult to anticipate due to a limit in the knowledge of its applications and development.

Although the evolution of technology has beneficial social effects, it certainly also generates negative effects, which are difficult to anticipate.

A similar situation occurs with the introduction of AI systems in medicine, whose long-term consequences are difficult to predict. The introduction of AI systems has had an impact on society, making the management of its unintended effects, as well as the development of an adequate legislative system that provides comfort and safety, a complex phenomenon [102].

In the development of AI fairness and biases, and the uniform and harmless application of AI systems, there is a need for collaboration between patients and advocacy groups, physicians, AI researchers, and AI developers to understand related challenges and concerns within the population. The establishment of professional societies that seek the removal of patients’ fears regarding the application of AI, that support their rights, and whose involvement in the development and implementation of these systems allows for increased transparency and trust, is very important [62].

Clear, generally accepted legislation for the mediation of medico-legal conflicts in cases of combined human and AI radiological diagnoses may reduce fears related to the consequences of applying AI systems in medicine—this is a desideratum.

## Figures and Tables

**Table 1:** The five ethical principles for AI use in healthcare [51].

Principle 1	Principle of Autonomy
Principle 2	Principle of Beneficence
Principle 3	Principle of Non-Maleficence—“primum non nocere”
Principle 4	Principle of Justice
Principle 5	Principle of Explicability—intelligibility and accountability

**Table 2:** The degree of AI involvement in radiological diagnostic reports [71].

	Level 0	Level 0.1	Level 1	Level 2	Level 3	Level 4
AI	No	CAD	Additional tool	Separation; pathologic and non-pathologic	Act autonomously	Autonomous action
Radiologist	Yes	Yes	Final validation	Validation; oversee pathologic cases	Complex case approval	No validation

No = No implication in the imaging report. Yes = Implications in the imaging report. Level 0 or traditional level: The activity is strictly performed by the radiologist. Level 0.1: The radiologist’s activity is assisted by computer-assisted diagnosis (CAD) through automatic lesion detection, but the final report is exclusively that of the radiologist. Level 1 (partial automation): AI works like an additional tool, but the radiologist supervises every action of the AI and formulates the final result. Level 2 (conditional automation): The AI system separates cases with normal results and pathological ones, with the latter being verified and validated by the radiologist. Normal cases are also described by the radiologist, using AI system prediction. Level 3 (high automation): The AI system acts autonomously to create and recommend differential diagnoses; complex cases need a radiologist’s approval and report. Level 4 (total automation): The AI operates autonomously to formulate a final diagnosis and recommendations for further imaging investigations. There is no final validation from a radiologist [82].

**Table 3:** Degree of implication of AI in diagnosis and liability [71].

Level of AI Implication	Liability
No AI	Radiologist
CAD	Radiologist
AI tool	Radiologist
AI assistant	Radiologist/Defective AI product
Independent AI	Controversial; AI legal personality?

## Data Availability

No new data were created or analyzed in this study.
